# Full-field Brillouin microscopy based on an imaging Fourier-transform spectrometer

**DOI:** 10.1038/s41566-025-01619-y

**Published:** 2025-02-20

**Authors:** Carlo Bevilacqua, Robert Prevedel

**Affiliations:** 1 Cell Biology and Biophysics Unit, European Molecular Biology Laboratory, Heidelberg, Germany; 2 Developmental Biology Unit, European Molecular Biology Laboratory, Heidelberg, Germany; 3 Epigenetics and Neurobiology Unit, European Molecular Biology Laboratory, Rome, Italy; 4 German Center for Lung Research (DZL), Heidelberg, Germany

**Keywords:** Optical spectroscopy, Microscopy

## Abstract

Brillouin microscopy is an emerging optical elastography technique that can be used to assess mechanical properties of biological samples in a three-dimensional, all-optical and hence non-contact fashion. However, the low cross-section of spontaneous Brillouin scattering produces weak signals that often necessitate prolonged exposure times or illumination dosages that are potentially harmful for biological samples. Here we present a new approach for highly multiplexed and therefore rapid spectral acquisition of the Brillouin-scattered light. Specifically, by exploiting a custom-built Fourier-transform imaging spectrometer and the symmetric properties of the Brillouin spectrum, we experimentally demonstrate full-field 2D spectral Brillouin imaging of phantoms as well as biological samples, at a throughput of up to 40,000 spectra per second, with a precision of ~70 MHz and an effective 2D image acquisition speed of 0.1 Hz over a ~300 × 300 µm^2^ field of view. This represents an approximately three-orders-of-magnitude improvement in speed and throughput compared with standard confocal methods, while retaining high spatial resolution and the capability to acquire three-dimensional images of photosensitive samples in biology and medicine.

## Abstract (web summary)

A Fourier-transform imaging spectrometer enables two-dimensional spectral Brillouin imaging at a throughput of up to 40,000 spectra per second over a 300 × 300 µm² field of view.

## Main

Mechanical properties of cells and tissues such as elasticity and viscosity are important parameters that have been shown to play crucial roles in determining biological function^1,2^; however, the standard techniques currently used to assess them exhibit intrinsic limitations such as requiring physical contact, being limited to surfaces, or poor resolution^3^.

**Fig. 1:**
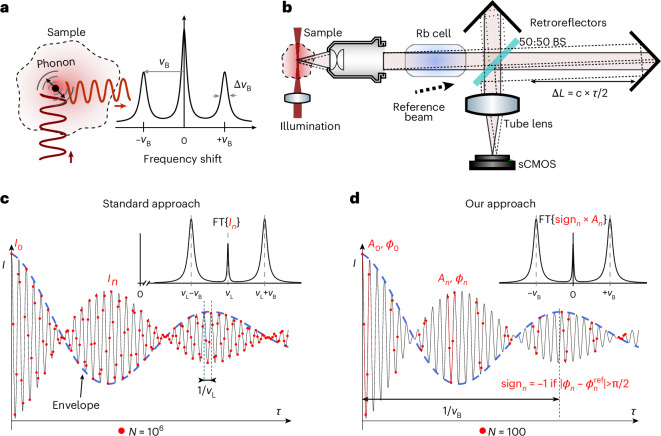
Principle of FTBM. **a**, In Brillouin scattering a small portion of the incident light (dark red, upwards-pointing arrow) interacts with thermal phonons intrinsically present in the sample. This gives rise to a scattered light (Brillouin) spectrum with two symmetrical peaks on the side of the main laser line with shift and width as indicated. B, Brillouin; *ν*_B_, Brillouin shift; Δ*ν*_B_, Brillouin linewidth. **b**, Conceptual schematic of FTBM: the sample is illuminated with a light-sheet (side-view only here); an objective lens collects the scattered light and a rubidium cell suppresses the elastically scattered light without affecting the Brillouin signal. After going through a Michelson interferometer, a tube lens forms the image of the sample on the sensor; a reference beam is also introduced to provide a reference for determining the optical phase (see Supplementary Note 2). sCMOS, scientific complementary metal–oxide–semiconductor. BS, beam splitter; *c*, speed of light. **c**,**d**, Comparison of sampling the interferogram (that is, the intensity *I* on the detector versus the time delay $\tau$ between the two arms of the Michelson interferometer) between standard Fourier-transform spectroscopy (**c**) and our approach (**d**). In the standard approach, the full interferogram is sampled according to the Nyquist–Shannon criterion, requiring ~10^6^ points (red dots) to achieve the necessary spectral resolution of ~500 MHz. *I*, intensity. **d**, In our approach the interferogram is only locally sampled, with much fewer points, to reconstruct the amplitude (*A*, blue dashed line) of the envelope, while the local phase (*ϕ*) is used to determine its sign; our approach requires only ~100 points. sign_*n*_, sign of the envelope. Insets: in both cases the full spectrum can be reconstructed by performing the Fourier transform of the samples; however, the spectrum is centred at the laser frequency ${\nu }_{\rm{L}}$ in the standard approach, whereas it is centred at zero in our approach.

In recent years, Brillouin microscopy^4,5^ has emerged as a non-destructive, label- and contact-free method that can probe the viscoelastic properties of biological samples with diffraction-limited resolution in three-dimensions. It relies on the interaction of light with spontaneous, thermally induced density fluctuations. This interaction gives rise to two additional peaks in the scattered light spectrum known as Stokes and anti-Stokes Brillouin peaks (Fig. 1a). The position of the peaks (Brillouin shift) and their linewidth (Brillouin width) are related to the elastic and viscous properties respectively^4^.

Regrettably, the spontaneous Brillouin scattering cross-section is weak, resulting in low scattering probabilities (10^–12^) that consequently necessitate long signal integration times and thus result in very slow imaging speeds, with typical measurement times of tens to hundreds of milliseconds for a single datapoint (that is, minutes to hours for 50–250 px^2^ two-dimensional images)^6–8^. Although recent work has made substantial progress by either: (1) multiplexing the data acquisition by illuminating and collecting spectra along an entire line^9–11^, or (2) relying on stimulated Brillouin scattering approaches^12–14^, overall imaging speed has remained far from fluorescence microscopy modalities, which lie in the range of microseconds per pixel. In particular, despite recent promising work in this direction^15,16^, a practical solution for 2D multiplexing, akin to light-sheet microscopy^17^, allowing for the capture of entire 2D Brillouin images simultaneously, currently does not exist.

In this work we present a new approach for Brillouin spectroscopy that is based on a custom Fourier-transform imaging spectrometer and an optimized sampling approach specifically designed for the high, subpicometre (that is, subgigahertz) spectral resolution required for Brillouin imaging. Importantly, the use of an imaging spectrometer enables full-field measurements of entire two-dimensional planes concurrently with array detectors (cameras), similar to a wide-field or light-sheet^17^ microscope. Although Fourier-transform imaging spectroscopy has found numerous applications in fields such as hyperspectral^18,19^ or Raman^20^ imaging, applications to Brillouin spectroscopy have so far been challenging due to the high spectral resolution required (subgigahertz), which in turn necessitates the acquisition of a large number of samples over a large range of interferometer delay positions (typically ~10^6^). Key to our approach is the realization that the symmetric properties of the Brillouin spectrum can be exploited to considerably (>10,000-fold) reduce the number of measurements to reconstruct a typical Brillouin spectrum. When combined with narrowband spectral filtering via atomic gas cells^21^, this allows for suppression of the dominating Rayleigh background, which in turn enables practical Brillouin imaging applications in biology and beyond. Overall, our approach—termed Fourier-transform Brillouin microscopy (FTBM)—provides a substantial speed increase for 2D Brillouin spectral imaging, as conventional cameras can record millions of pixels simultaneously, with corresponding reduction in overall imaging time and light exposure, which is highly advantageous for various (biological) imaging applications. We demonstrate the effectiveness and capabilities of our FTBM to record Brillouin microscopic imagery at high spatial and temporal resolution by imaging heterogeneous phantoms as well as live zebrafish larvae.

## Results

### Principle of the FTBM approach

The principles underlying Brillouin microscopy, as well as our particular approach, are outlined in Fig. 1. Fourier-transform spectroscopy is an established technique for spectral characterization, and relies on measuring the optical power at the output of an interferometer as a function of the optical delay. Applying a Fourier transform to the acquired data retrieves the power spectral density as a function of optical frequency.

Specifically, in case of a Michelson interferometer (Fig. 1b), the intensity at the output of the interferometer is given by:

$$I\left(\tau \right)={\left\langle {\left|E\left(t\right)/\sqrt{2}+E\left(t-\tau \right)/\sqrt{2}\right|}^{2}\right\rangle }_{t}={I}_{0}+{\left\langle E\left(t\right)E\left(t-\tau \right)\right\rangle }_{t}$$

where $E\left(t\right)$ is the optical electric field, ${\left\langle \cdot \right\rangle }_{t}$ indicates the average over the integration time of the detector, $\tau =\frac{2\Delta L}{c}$ is the time delay between the two arms of the Michelson interferometer (Δ*L* is the length difference between the two arms of the Michelson), and ${I}_{0}={\langle {\left|E\left(t\right)\right|}^{2}\rangle }_{t}$.

The ${\left\langle E\left(t\right)E\left(t-\tau \right)\right\rangle }_{t}$ term represents the autocorrelation function for the electric field and, according to the Wiener–Khinchin theorem, corresponds to the Fourier transform of the optical spectrum. Hence, Fourier-transform spectroscopy determines the optical spectrum from the interferogram $I\left(\tau \right)$. Practically, one samples $I\left(\tau \right)$ at discrete intervals, ${\tau }_{n}=\frac{2{\Delta L}_{n}}{c}$ (Fig. 1c). The sampling rate and the number of samples required to properly reconstruct the spectrum from $I\left(\tau \right)$ can be easily found by considering the Nyquist–Shannon sampling theorem and the properties of the discrete Fourier transform. Specifically, the required sampling rate is at least twice the maximum optical frequency present in the optical spectrum, that is $\delta {\tau }_{n} < \frac{{\lambda }_{\min }}{2c}$ → $\delta {\Delta L}_{n} < \frac{{\lambda }_{\min }}{4}$. In turn, the maximum time delay ${\tau }_{\max }$ determines the spectral resolution $\Delta \nu$ achievable. The required number of samples thus becomes:

1 $$N=\frac{{\tau }_{\max }}{\delta {\tau }_{n}} > \frac{1/\Delta \nu }{{\lambda }_{\min }/(2c)}=\frac{2c}{\Delta \nu \times {\lambda }_{\min }}$$

For measurements of a typical Brillouin spectrum, for example, at a wavelength of ${\lambda }_{\min }$ = 780 nm, for which a high spectral resolution of $\Delta \nu$ = 0.5 GHz is normally required, the overall number of samples becomes *N* > 10^6^. If we consider 10–100 ms integration time, which is a typical value for acquiring Brillouin spectra from biological samples^22^, this practically entails between ~3 and ~30 h of acquisition time. Past work has explored the use of compressive sensing approaches to reduce the number of required samples in Raman Fourier-transform spectroscopy, and has found an approximately fourfold improvement by exploiting the sparsity of the Raman spectrum^23^. By contrast, in our work we show that, by exploiting the symmetry of the Brillouin spectrum, we can reduce the required number of samples by >10^4^-fold, as outlined briefly below (see Supplementary Note 1 for further details).

The main realization is that for a symmetric band-limited spectrum, the interferogram $I\left(\tau \right)$ consists of a fast-oscillating term at the central frequency enclosed by a slowly varying envelope that contains the full spectral information. The optical spectrum can therefore be measured by locally determining the envelope of the interferogram. This can be achieved by sampling the interferogram at the central frequency (laser optical frequency, ${\omega }_{\rm{L}}$) with only few points ${N}_{\rm{L}}$ (in principle, ${N}_{\rm{L}}=3$ points are sufficient if only the amplitude, phase and offset need to be recovered), from which the signed amplitude of the oscillation $A\left(\tau \right)$ can be determined (Fig. 1d and Supplementary Note 2).

Specifically, the optical spectrum can be reconstructed by taking the Fourier transform of $A\left(\tau \right)$, with the only difference that it will be centred at 0 instead of at the laser frequency ${\omega }_{\rm{L}}$.

Consequently, we only need to apply the sampling conditions to the function $A\left(\tau \right)$ instead of to the full interferogram $I\left(\tau \right)$. In particular if the bandwidth of the spectrum is upper-limited by ${\omega }_{\max }+\Omega$, then $A\left(\tau \right)$ can be reconstructed by sampling it at $\delta {\tau }_{n} < \frac{\uppi }{{\omega }_{\max }+\Omega }$.

2 $$N={N}_{\mathrm{L}}\frac{{\tau }_{\max }}{\delta {\tau }_{n}} > {N}_{\mathrm{L}}\frac{1/\Delta \nu }{\uppi /({\omega }_{\max }+\Omega )}={N}_{\mathrm{L}}\frac{{\omega }_{\max }+\Omega }{\uppi \Delta \nu }$$

Note that $${\omega }_{\max }$$ represents the maximum frequency in the spectrum that can be reconstructed unambiguously. For Brillouin spectra measured from typical biological samples at 780 nm, $${\omega }_{\max }+\Omega$$$$\lesssim 2\uppi \times 7 \, {\rm{GHz}}$$, and therefore $$N > {N}_{\rm{L}}\frac{7 \, {\rm{GHz}}}{\Delta \nu }\approx 30$$, which is an at least 10^4^-fold reduction compared with the standard approach requiring *N* > 10^6^ samples, as calculated from equation (1).

We note that our derivation is not limited to Brillouin spectroscopy and can be applied to any symmetric spectrum. In general, the reduction in the number of required points $r$ is given by the ratio of equations (1) and (2):

3 $$r=\frac{2c}{\Delta \nu \times {\lambda }_{\min }}\frac{1}{\left({N}_{\rm{L}}\frac{{\omega }_{\max }+\Omega }{\uppi \Delta \nu }\right)}=\frac{2\uppi c}{{\lambda }_{\min }{N}_{\rm{L}}\left({\omega }_{\max }+\Omega \right)}\approx \frac{{\omega }_{\rm{L}}}{{N}_{\rm{L}}\left({\omega }_{\max }+\Omega \right)}$$

which corresponds to the ratio between the optical frequency, ${\omega }_{\rm{L}}$, and the frequency range ${\omega }_{\max }+\Omega$ over which one aims to reconstruct the spectrum.

Finally, we note that the finite linewidth of the Brillouin peaks ${\Delta \nu }_{\rm{B}}$ causes an exponential decay of the amplitude (see Supplementary equation 1.5), thus effectively setting a practical upper limit to the optical path difference that needs to be scanned. Further increasing $\tau \propto \Delta L$ will not increase the spectral resolution but predominantly sample noise.

### Experimental performance characterization

**Extended Data Fig. 1:**
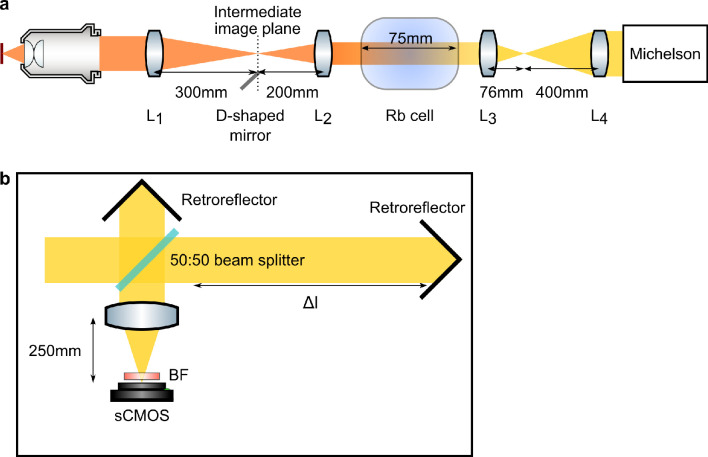
Optical design of the FTBM. **a**, Conceptual schematic of the optical layout. An objective (not shown) generates a light sheet (red line on the left). The scattered light is imaged into an intermediate image plane by an identical objective (40x, 0.8NA, Nikon) and a 300 mm plano-convex lens. A D-shaped mirror in the intermediate image plane is used to introduce a reference beam in the same optical path. A ^87^Rb vapour cell is used to filter the unwanted elastically scattered light. A combination of lenses subsequently prepares the light to be input into the Michelson interferometer (see Methods for more details). **b**, Optical schematic of the Michelson interferometer consisting of a 50:50 beam splitter and 2 hollow retroreflectors. A 250 mm tube lens generates the image of the sample on the camera. BF, bandpass filter to suppress stray light (centred at 780 nm with a bandwidth of FWHM = 10 nm).

To demonstrate the feasibility of our subsampling approach for experimental Brillouin measurements, we designed and built a custom Fourier-transform interferometer and coupled it to an inverted SPIM microscope^9^ (Methods and Extended Data Fig. 1). The Fourier-transform interferometer consists of a Michelson interferometer, which is placed in the infinity space of the imaging system (Fig. 1b), and uses corner-cube reflectors for high stability of operation^24^. A ^87^Rb vapour cell, placed before the Michelson interferometer, is used to suppress the elastically scattered light while not affecting the Brillouin signal^9^. A reference beam, taken from the laser, is introduced in the interferometer to aid in determining the sign of the amplitude (Methods and Supplementary Notes 2–4).

**Fig. 2:**
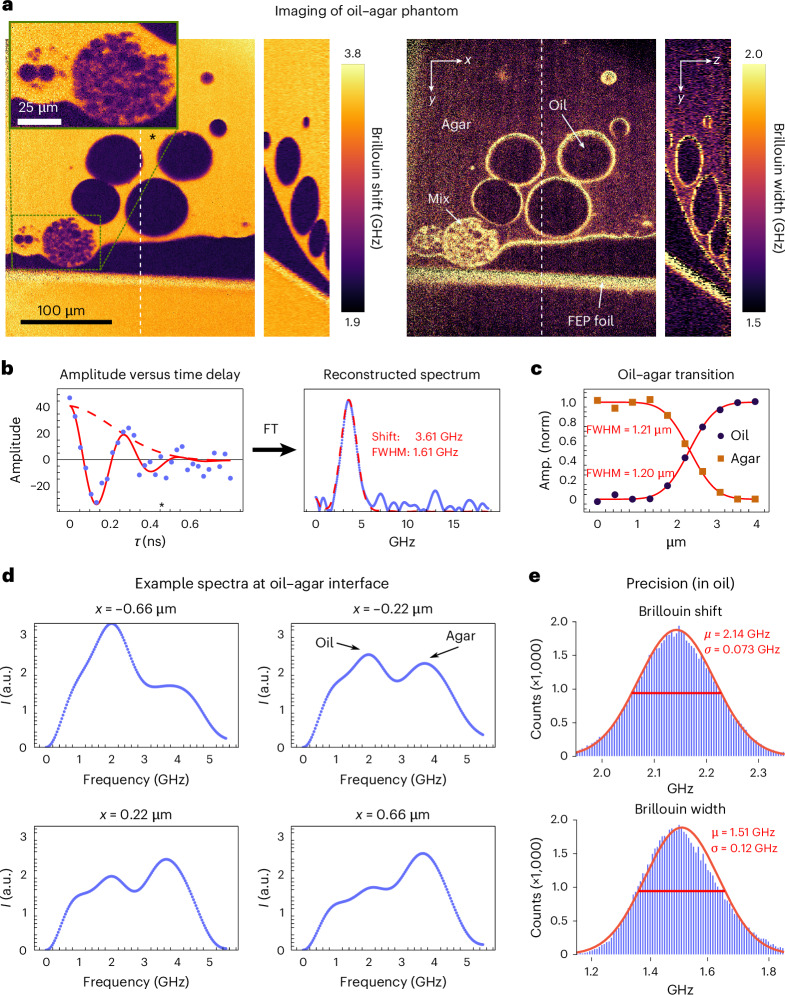
Experimental characterization of the FTBM. **a**, Three-dimensional imaging from a material phantom, consisting of oil droplets in agar (see Methods), both for the Brillouin shift (left) and Brillouin linewidth (right). The field of view of the imaged volume is 273 × 323 × 72 µm^3^, corresponding to 621 pixels × 735 pixels × 36 pixels; the white vertical dashed line indicates the plane shown in the orthogonal view; the asterisk indicates the position of the spectrum shown in **b** (see also Supplementary Video 1). **b**, Example interferogram (left) and corresponding Fourier transform (FT, right). Note that the absolute value of the plotted amplitude corresponds to actual number of photoelectrons detected by the camera. The solid red line in the left panel shows the fit with Supplementary equation (5.1) to experimental interferogram data, whereas the dashed red line indicates its amplitude decay (which defines the linewidth). The dashed red line in the right panel indicates the FT of the interferogram data in the left panel (and not a fit performed on the FT spectrum). **c**, Plot of the amplitude (normalized) of the oil component (at 2.15 GHz) and the agar component (at 3.53 GHz) through the edge of a large bead (taken from the stack shown in **a**). Each point is calculated by fitting the spectrum (taken as the average of nine adjacent points) with two Gaussian peaks centred at the oil and agar Brillouin shifts, respectively. The solid lines show a fit with an erf function, from which the FWHM is derived. **d**, Example spectra (average of six adjacent points) along the transition between oil and agar, showing the two peaks at the oil and agar Brillouin shifts. Note that it is a different transition than the one shown in **c**. **e**, Histograms representing the precision of the Brillouin shift (top) and width (bottom) for a large oil region taken from the stack shown in **a**. The solid red curves represent Gaussian fits with the mean and s.d. calculated from the data, as indicated by the red text. Source data

**Extended Data Fig. 2:**
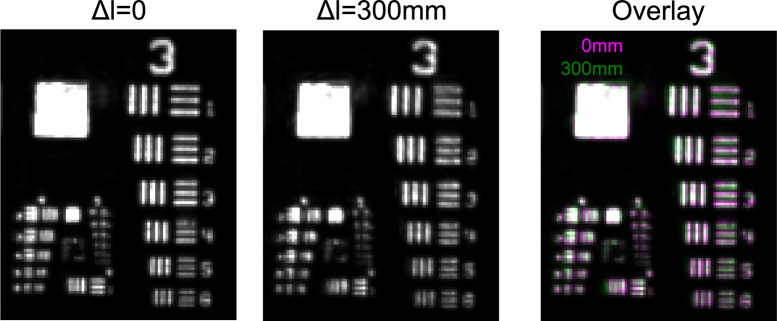
Imaging performances through the FT spectrometer. Images of a 1951 USAF resolution target, placed in the intermediate image plane, as seen through the FT spectrometer when the fixed arm is blocked and the moving arm is positioned at ΔL = 0 and ΔL = 300 m. We note that group 3 element 6 can still be resolved in both cases, corresponding to a resolution on the sample plane of 0.58μm. The overlay image highlights that, even with an increase of optical path of 2ΔL = 0.6 m, the images are still overlapping to a high degree which is essential to generate proper interference between the two interferometer arms.

To characterize the performance of our FTBM system, we imaged a heterogeneous phantom consisting of oil beads embedded in agar over a ~300 × 300 µm^2^-wide field of view (Fig. 2 and Supplementary Video 1). The Brillouin shift and width for each point were extracted by fitting the interferogram with Supplementary equation (5.1) (derived in Supplementary Note 5), which takes into account the numerical aperture broadening^25^. An example interferogram with the corresponding fit is shown in Fig. 2b. The Fourier transform of the interferogram is also shown to highlight the quality of the recovered optical spectrum. The spatial heterogeneity of the sample, especially the presence of agar inclusions inside the oil (Fig. 2a, inset), showcases the high spatial and mechanical resolution of our FTBM system on micrometre length scales. Specifically, we quantified the spatial resolution by evaluating a sharp transition between agar and oil at the edge of a bead. To this end, we performed a Gaussian double-peak fit on the reconstructed spectra along the transition and plotted the amplitude of the oil and agar component (Fig. 2c). A fit with an ‘erf’ function shows a consistent full-width at half-maximum (FWHM) of ~1.2 μm for the two curves. Figure 2d shows the spectra along a different (less sharp) transition, where the oil and agar peaks are clearly visible with varying amplitudes. To further corroborate the high imaging resolution and degree of spectrometer alignment, we placed an USAF resolution target in the intermediate image plane (as defined in Extended Data Fig. 1) and imaged it through the Michelson interferometer at different positions of the scanning arm while blocking the reference arm. This demonstrates that the resolution and magnification of the optical system can be preserved even with a $2\times \Delta L$ = 600 mm increase in optical path (Extended Data Fig. 2).

**Extended Data Fig. 3:**
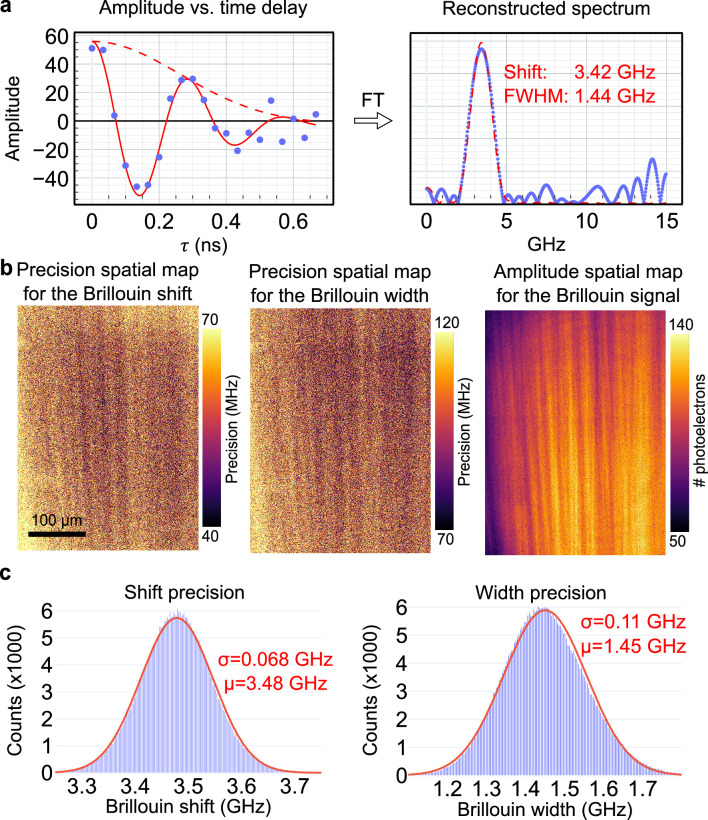
Quantification of the spectral precision of the FTBM in water. **a**, Example interferogram (left) and corresponding Fourier Transform (FT, right) of distilled water. Note that the absolute value of the plotted amplitude corresponds to actual number of photoelectrons, as detected by the camera. Solid red line in the left panel shows the fit (with the function S5.1) to experimental interferogram data while the dashed red line indicates its amplitude decay (which defines the linewidth). The dashed red line in the right panel indicates the Fourier Transform of the fitted interferogram data on the left (and not a fit performed on the FT spectrum). **b**, Spatial precision map for the Brillouin shift and width of distilled water (553x745 px^2^). The value (precision) of each pixel is calculated as the standard deviation of 15 replicates (images of water) acquired with the parameters described in the methods (5x20 samples, 100 mm total travel range), ~70 mW optical power on the sample and 100 ms integration time for each sample of the interferogram (that is 10 s of cumulative exposure time for each image). We note that the slight spatial variation in precision is a direct result of the non-homogenous light-sheet illumination intensity which in turn affects the amplitude of the signal as shown on the right. **c**, Histogram of the Brillouin shift and width for an entire single frame in panel a,b. The solid red curve represents a Gaussian with the mean and standard deviation calculated from the data as indicated.

The throughput of our approach can be calculated by considering that a single 2D image from the stack shown in Fig. 2a consists of 621 × 735 px^2^ (spectra), reconstructed from 155 individual scanning arm positions, with 100 ms exposure time for each, for a total data integration time of 15.5 s (Methods). This corresponds to a throughput of ~30.000 spectra per second. We note that in our current implementation the actual acquisition time is slightly longer, due to stage movement, but it can straightforwardly be improved by using a faster (direct drive) stage with speeds of >1 m s^–1^. Finally, to characterize the spectral precision, we generated a histogram for the Brillouin shift and width from a large homogeneous oil region (Fig. 2e); we found a standard deviation of 73 MHz and 120 MHz, respectively, in line with theoretical simulations (see the next section). To show that the shift precision is homogeneous across the entire FOV, we plotted a spatial map of the precision for water (Extended Data Fig. 3), and obtained shift and width precisions of 68 MHz and 110 MHz, respectively, consistent with the results obtained for oil in the heterogeneous phantoms.

We highlight that, in principle, the same set-up, albeit without undersampling, could be used for Raman imaging, providing complementary chemical information on the sample that can aid in further interpretation of the measured mechanical properties^26,27^.

### Numerical performance evaluation

Next, we sought to compare our experimental results with theory to evaluate how the different experimental parameters might affect the performance of our measurements. To this end, we devised the following numerical simulation framework: we assumed a Brillouin spectrum of water broadened due to the finite collection NA^25^ and computed the Fourier-transform interferometer transfer function (see Supplementary Note 5 for derivation) in terms of the number of detected photo(electro)ns on the camera, ${N}_{\rm{detect}}$, as a function of the optical delay $\tau$:

4 $$2{N}_{\rm{detect}}={N}_{\rm{ASE}}+{N}_{\rm{elas}}+{N}_{\rm{Br}}+\left[{N}_{\rm{elas}}+{N}_{\rm{Br}}\times {\rm{A}}(\tau )\right]\times \cos \left({\omega }_{L}\tau \right)$$

where ${\rm{A}}(\tau )$ is defined in Supplementary equation 5.1, ${N}_{\rm{ASE}}$ is the number of photons for the amplified spontaneous emission (which is assumed to be completely incoherent and therefore not contributing to the interference term $\cos \left({\omega }_{L}\tau \right)$); ${N}_{\rm{elas}}$ is the number of elastically scattered photons; and ${N}_{\rm{Br}}$ is the number of Brillouin-scattered photons at the input of the Michelson interferometer. A factor of two is introduced to take into account that the average number of photons at the output of the Michelson interferometer is half of the one at the input. Supplementary Note 6 provides guidance in converting ${N}_{\rm{detect}}$ to experimental parameters such as input optical power and/or dwell time. As outlined below, we further considered various sources of experimental noise and imperfections to make these simulations as realistic as possible. We then locally sampled this interferogram with *n* = 5 samples, spaced by a change in optical path length (OPL) of *λ*/4 = 195 nm, and reconstructed the local amplitude and phase by fitting a cosine function with fixed frequency (given by 4π/*λ*). The same procedure is repeated after increasing the OPL iteratively by 20 larger steps of 10 mm to estimate the overall interferogram’s envelope. The Brillouin shift and linewidth are then determined by least-square fitting of the so-obtained datapoints with Supplementary equation (5.1).

**Fig. 3:**
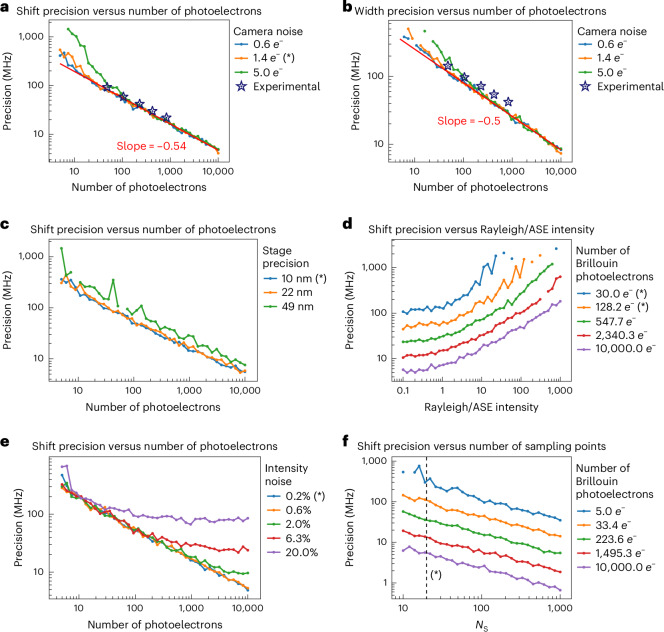
Numerical simulation of the FTBM performance. **a,b**, Shift (**a**) and width (**b**) precision as a function of the number of detected photons at varying read-out noise levels of the camera (reported as s.d.). The stars indicate experimentally measured values in water (see Methods) and confirm the quantitative validity of our simulations. **c**, Shift precision as a function of the number of detected photons at varying levels of Fourier-transform interferometer stage precision (reported as s.d.). **d**, Shift precision as a function of Rayleigh intensity at varying numbers of detected photons. Note that the effect of the ASE intensity on the precision is similar, which is why we plot them on the same scale. **e**, Shift precision as a function of the number of detected photons at varying intensity noise (reported as s.d.). **f**, Shift precision as a function of the number of sampling points (*N*_S_) at varying numbers of detected photons per sampling step (see Methods for details). The precision is calculated as the s.d. of 100 replicates. The reported number of photoelectrons corresponds to the maximum intensity of the Brillouin photons on the photodetector at OPL = 0. The asterisks (*) in the legends indicate the experimental parameters used in our implementation. The red lines in **a**, **b** and **f** are linearly fit (in the log–log plots) to the right half of the simulation curves. Source data

Our numerical simulations are generally in good agreement with experimental measurements—both of which exhibit the expected shot-noise-limited performance (log–log slope of –0.5; Fig. 3). We also found that a high level of precision in the Brillouin shift (<20 MHz) and width (<50 MHz) estimates can be obtained with only ~1,000 detected photons when scanning over 20 discrete OPL steps (Fig. 3a,b). Importantly, our simulations provide valuable scaling laws of the expected performance of our FTBM system with respect to the detected signal intensity as well as various sources of experimental error, including camera read-out noise (Fig. 3a,b), stage precision (Fig. 3c), as well as Rayleigh, amplified spontaneous emission (ASE), and intensity noise (Fig. 3d,e). Note that, in practice, ASE intensity levels can be highly dependent on the sample and its amount of elastic scattering. Finally, the simulations provide guidance towards the expected precision as a function of the OPL sampling steps and overall signal level (Fig. 3f). Here we note that the precision is only dependent on the overall number of photons detected, and independent of the number of sampling points over which those photons are spread. Overall, the results show that state-of-the-art Brillouin measurements (precision < 10 MHz) can be expected with <3,000 collected photoelectrons, even with consumer-grade cameras or translation stages and assuming realistic Brillouin signal levels. Finally, we note that we only considered a single noise source (in addition to shot noise) in the panels of Fig. 3, whereas, experimentally, multiple sources are probably present at the same time. How their combined effect influences the precision is complex, however, for typical experimental parameters shot noise is dominant (indicated by the –0.5 slope in Fig. 3). Supplementary Table 1 shows examples of the combined effect of multiple noise sources in the regime of low photons numbers.

### Mechanical imaging in biological samples

**Fig. 4:**
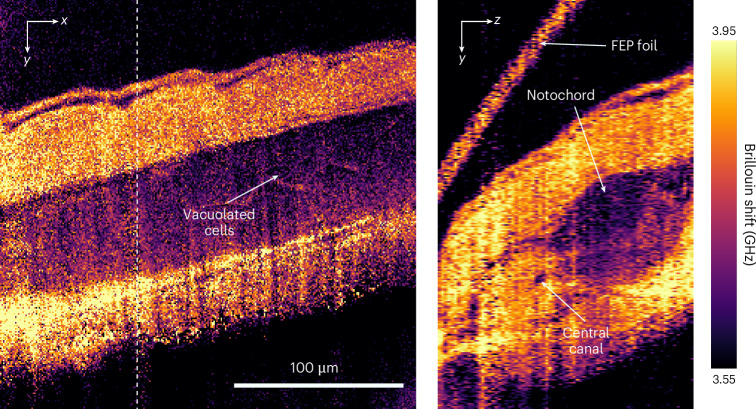
FTBM imaging of a live zebrafish notochord. The white dashed line indicates the position of the axial cross-section shown on the right. Anatomical features of the notochord region such as vacuolated cells and the central canal are visible in the lateral as well as axial views. The image was acquired 2 days post-fertilization. The total illumination power was 56 mW; 76 planes were acquired for a total FOV of 245 × 240 × 151 µm^3^, which corresponds to 279 pixels × 273 pixels × 75 pixels.

After characterizing the optical performance, we tested the capabilities of our FTBM to image real-world biological samples. For this we acquired FTBM images over an FOV of 245 × 240 × 151 µm^3^ in the tail region of a live zebrafish larvae at 2 days post-fertilization (Fig. 4). With 56 mW of total illumination power, corresponding to 0.7 µW per pixel, we obtained high-signal-to-noise-ratio Brillouin-shift images of the region surrounding the notochord, from which known anatomical landmarks such as muscle tissue, vacuolated cells and the central canal can be clearly discerned. Compared with point-scanning Brillouin microscopy implementations such as confocal or stimulated Brillouin scattering, we note that the large thickness of the light-sheet combined with a twofold-lower spatial resolution and sampling of FTBM leads to less apparent details and blurring. Furthermore, small artefacts become present on the far side of the tissue due to a less confined illumination laser there and possible refraction. Nevertheless, our results confirm the in-principle suitability of FTBM for biological imaging applications. Specifically, we highlight that the required illumination energy to reconstruct the Brillouin spectrum in FTBM is only 11 µJ per pixel, which compares very favourably to confocal^6–8^ Brillouin microscopy (~0.5–5 mJ per pixel), and is similar to line-scan^9,10^ (~10 µJ per pixel) Brillouin microscopy implementations that were used to acquire similar biological samples, albeit at higher shift precision of 20 MHz. However, we further note that the actual power density per pixel per spectrum is only 0.7 µW per pixel for FTBM, but 5–50 mW per pixel for confocal, and 0.1 mW per pixel for line-scan Brillouin microscopy. As both power density and total energy need to be limited to avoid detrimental effects of photodamage, FTBM will be specifically advantageous for bioimaging applications of photosensitive samples.

## Discussion

In this work we demonstrated a new approach and key working principle for fast, high-resolution full-field 3D imaging of mechanical properties based on a Fourier-transform Brillouin spectrometer. We demonstrate its ability to visualize mechanical properties of simple phantoms, as well as real-world biological samples at much higher speed and overall throughput compared with alternative Brillouin imaging modalities^6–11^. In particular, compared with line-scan Brillouin microscopy, FTBM achieves a >100-fold reduction in illumination density, albeit with a slightly lower spectral precision (~70 MHz versus 20 MHz). Better precision can be obtained by tradeoffs in input energy and acquisition time, as outlined in Fig. 3 and Supplementary Note 6. In terms of effective pixel time (that is, the number of spectra measured per second), FTBM achieves acquisition times of ~25 µs per pixel, similar to a recent realization of stimulated Brillouin scattering gain microscopy^28^, which, however, only probes the Brillouin gain at a single frequency of interest. By contrast, here we can reconstruct a full spectrum that can be composed of multiple peaks or modes over a 15 GHz range on the same timescale. This substantial overall boost in performance is due to the highly multiplexed measurements afforded by the full-field detection, and could be easily further improved by exploiting the full chip of a large sensor sCMOS cameras. Furthermore, our FTBM approach even achieves high spectral precision with consumer-grade instrumentation such as higher-read-noise cameras (due to the Fellgett’s advantage) or less precise translation stages (Fig. 3a–c). We expect these facts to facilitate further uptake by the bioimaging community and aid in overall dissemination. Moreover, in contrast to current spontaneous or stimulated Brillouin microscopy methods, the frequency calibration of the reconstructed spectrum does not rely on a known material or an external frequency reference, but instead is determined by the accuracy of the motorized stage, which can easily exceed 0.1%, translating to a frequency accuracy of <5 MHz. Another distinct advantage of our Fourier-transform-based approach is the fact that optimization of the interferogram sampling enables straightforward modification of the sensed spectral frequency range while retaining the spectral resolution. This is unlike common Brillouin spectrometers based on, for example, VIPAs, in which larger spectral ranges typically come at the expense of lower spectral resolution. Indeed, in our work we have used different interferogram sampling settings for water and heterogeneous samples (see Methods). For example, in the case of water, which has a single, well-defined Brillouin peak, and which is typically used to compare Brillouin spectrometer performances, we could indeed achieve an overall throughput of more than ~40,000 spectra per second with 1.4 GHz resolution (note that in our geometry the Brillouin peak of water has an FWHM of 1.5 GHz) over a range of 15 GHz.

Current limitations of our approach are shared with other modalities that use separate excitation and detection paths, such as light-sheet microscopy, namely, that optical sample accessibility from two sides must be ensured, and optical refraction effects between the sample and medium can lead to artefacts. The latter could in principle be mitigated by refractive index matching^29^ or adaptive optics methods^30^. Alternatively, merging our approach with axial plane microscopy realizations^31^ would provide a higher scattering angle (180° versus 90°), easier sample mounting or accessibility, and less susceptibility to refraction effects. Further thinning of the light-sheet illumination (currently ~10 µm) to match it to the axial resolution of the objective, as well as general optimization of the Fourier-transform spectrometer’s optical design, are further expected to improve overall spatial resolution and image quality. At present, a practical limitation is the maximum available laser power (<70 mW), which limits the power density at sample plane, due to the width of the light-sheet and, generally, the large FOV afforded by our FTBM. We also note that ASE filtering of the illumination laser is critical to obtain high-signal-to-noise-ratio spectral recordings. Here, although the elastic (Rayleigh) background can be effectively (>80 dB) suppressed by the rubidium cell, the ASE overlaps spectrally with the Brillouin frequency of interest and therefore needs to be adequately filtered out in the illumination arm before reaching the sample.

Finally, we highlight that our approach to undersampling in Fourier-transform-based spectral imaging is in principle of general nature and can be applied to any symmetric spectrum; however, we have only confirmed it here for symmetric Brillouin spectra. Moreover, without undersampling, the same set-up with appropriate filters should also be able to acquire concurrent Raman spectra together with the Brillouin signal^26,27^, which can be used to correlate the mechanical properties with the local chemical composition of biological specimens to obtain a deeper understanding of the measured mechanics. Going forward, we expect our method to find numerous applications in fast and/or high-throughput Brillouin-scattering-based imaging applications both in and outside of biology.

## Methods

### FTBM set-up and design

The Brillouin illumination and objective configuration is similar to that described in ref. ^9^, consisting of two identical objectives (Nikon, 40×, 0.8 numerical aperture, MRD07420, water immersion) mounted in an inverted V-shaped geometry. To generate the light-sheet, a plano-convex lens (354330-B, Thorlabs, focal length *f* = 3.1 mm) was used to collimate the laser out of a polarization-maintaining fibre (P3-780PM-FC-2, Thorlabs) to a beam diameter of 0.48 mm 1/e^2^ (theoretical). A cylindrical lens (LJ1310L1-B, Thorlabs, *f* = 4.01 mm) focuses the light onto a plane conjugated to the back focal plane of one of the two objectives. The resulting light-sheet has a theoretical width of 598 µm (1/e^2^), length of 287 µm (Rayleigh range) and thickness of 10 µm (1/e^2^).

On the detection side, a plano-convex lens (LA1256-B, Thorlabs, *f* = 300 mm) generates an intermediate image (with an effective magnification of 60×). Here a D-shaped mirror it is used to introduce a reference beam at the edge of the field of view. The reference, taken out of the 1% port of a fibre beam splitter (PN780R1A2, Thorlabs), passes through a fibre acousto-optic modulator (Brimrose, TEM-250-50-10-780-2FP). The acousto-optic modulator allows for the light intensity to be adjusted and also introduces a 250 MHz frequency shift to reduce the absorption from the rubidium cell and thus avoid the complete suppression of the main laser line (note that the change in wavelength is sufficiently small to not cause a substantial phase-shift over the full travel range of the stage). Two plano-convex lenses (catalogue nos 39–150, Edmund, *f* = 30 mm and catalogue nos 39–152, Edmund, *f* = 100 mm) collimate and focus the light, respectively, on the D-shaped mirror in the intermediate image plane. Neutral density filters, with a total OD 7, reduce the intensity of the laser so that it can be acquired at the same time of the Brillouin signal without saturating the camera.

A plano-convex lens (LA1979-B-ML, Thorlabs, *f* = 200 mm) recollimates the light from the intermediate image plane, bringing it back to infinity space. There a 75-mm-long ^87^Rb cell (SC-RB87-(25 × 75-Q)-AR, Photonics Technologies) absorbs the elastically scattered light while transmitting the Brillouin signal (for the beads phantoms and zebrafish sample a 150-mm-long cell was used instead). A beam expander composed of a *f* = 76 mm (catalgue nos 49–794, Edmund) and a *f* = 400 mm (LA1725-B-ML, Thorlabs) lens expand the beam to 28 mm, thus reducing the angles inside of the Michelson interferometer.

The Michelson consists of a 50:50 beam splitter (catalogue no. 47–572, Edmund) and two retroreflectors (HM-15-1E, PLX)—one of which is mounted on a long travel range motorized stage (CLL42, SmarAct). After the Michelson interferometer, a tube lens (*f* = 250 mm) forms the image of the sample on an sCMOS camera (C11440-22CU, Hamamatsu). A bandpass filter (FBH780-10, Thorlabs) in front of the camera suppresses any unwanted background stray light.

### Imaging

For the imaging of the phantom (Fig. 2), the total power on the sample was 56 mW and the exposure time was 100 ms. We sampled the interferogram with five fine steps (${N}_{\rm{L}}$) of $\Delta L$ = 97.5 nm (OPL = 195 nm) and 31 coarse steps (${N}_{\rm{S}}$) of $\Delta L$ = 4 mm (OPL = 8 mm), corresponding to an accessible frequency range of 0–18.75 GHz with a resolution of 1.2 GHz. The parameters are identical for the zebrafish image in Fig. 4, with 2× binning applied before data reconstruction.

For the five experimental datapoints in Fig. 3a,b (and Extended Data Fig. 3), the total optical power on the sample (water) and the exposure time per stage position are: (33 mW, 100 ms), (70 mW, 100 ms), (136 mW, 100 ms), (168 mW, 150 ms), (168 mW, 300 ms). To sample the interferogram, five fine steps (${N}_{\rm{L}}$) of $\Delta L$ = 97.5 nm (OPL = 195 nm) and 21 coarse steps (${N}_{\mathrm{S}}$) of $\Delta L$ = 5 mm (OPL = 10 mm) were taken, corresponding to an accessible frequency range of 0–15 GHz with a resolution of 1.4 GHz. The data shown in Figs. 2a and 3a,b were reconstructed by fitting the interferogram with the function in Supplementary equation (5.1), assuming an effective NA of ~0.47 (see Supplementary Note 5). The data shown in Fig. 4 were reconstructed by fitting a Gaussian function to the spectra in frequency domain. The precision in Fig. 3a,b and Extended Data Fig. 3 is determined by acquiring 15 images of water, calculating the standard deviation and taking an average of a central region of the resulting image. The same oil–agar phantom shown in Fig. 2a was also imaged with a confocal, 660 nm double-VIPA Brillouin set-up. When extrapolating to 780 nm and 90° scattering geometry, we found an equivalent average shift of 2.27 and 3.59 GHz for oil and agar, respectively. This agrees well with the measurements performed with FTBM.

### Numerical simulations

The numerical simulation was performed in Python v.3.11.7, numpy v.1.26.4 and scipy v.1.11.4. The raw interferogram was generated using equation (4), with sampling performed with $${N}_{\rm{L}}=5$$ fine steps and $${N}_{\rm{S}}=20$$ coarse steps. Furthermore, the following noise sources were considered: shot noise was added to the simulated interferogram data by drawing each individual sample from a Poisson distribution having an expected value given by $${N}_{\rm{detect}}$$. Camera noise was subsequently added by drawing samples from a normal distribution, with the width reported in the legend of Fig. 3a,b. The stage precision in Fig. 3c was taken into account by drawing the stage position from a normal distribution with width reported in the legend and represents typically achievable values. The intensity noise in Fig. 3e was added before the shot noise by drawing samples from a normal distribution with the sigma given by the percentage reported in the legend multiplied by the intensity. The sampling points reported on the *x*-axis of Fig. 3f correspond to $${N}_{\rm{S}}$$, with the total OPL (given by $$2{N}_{\rm{S}}\times{\delta \Delta L}_{n}$$), kept constant at 200 mm. The number of photoelectrons reported on the axes in Fig. 3 equal the photons detected per sampling step. Thus, $${N}_{\rm{total}}=$$$$N_{\rm{detect}}\times {N}_{\rm{S}}\times {N}_{\rm{L}}$$ can be used to obtain the total number of photons.

### Sample preparation

The phantom was prepared by mixing oil (Immersol W2010, Zeiss) with 0.8% agar (with the addition of fluorescein to make it fluorescent) and keeping it in a warm ultrasound water bath during polymerization, so that some beads formed at the oil–agar interface.

Two-days-post-fertilization zebrafish larva from a citrine endogenously labelled α-catenin line, Gt(ctnna-citrine)ct3a, was used in Fig. 4. 1-Phenyl 2-thiourea was added at 0.003% concentration shortly after fertilization to avoid pigmentation. The fish was placed inside the imaging chamber in E3 medium. The sample is separated from the immersion liquid of the objectives (water) by a 12.7-µm-thick FEP foil (refer to ref. ^9^ for further details).

Animal work in this research was performed at the European Molecular Biology Laboratory (EMBL). All animal care and procedures performed in this study conformed to the ‘EMBL Guidelines for the Use of Animals in Experiments’ as outlined in EMBL Internal Policy 65 (‘EMBL policy on the protection of welfare of animals used for scientific purposes’) and were reviewed and approved by EMBL’s ‘Institutional Animal Care and Use Committee’.

## Online content

Any methods, additional references, Nature Portfolio reporting summaries, source data, extended data, supplementary information, acknowledgements, peer review information; details of author contributions and competing interests; and statements of data and code availability are available at 10.1038/s41566-025-01619-y.

## Supplementary information

Supplementary Information Supplementary Notes 1–6 and Table 1.

Supplementary Video 1 Three-dimensional imaging of a heterogeneous material phantom: there is an animation of the image stack shown in Fig. 2 for both the Brillouin shift (left) and linewidth (right). The current *z*-position (depth) is indicated at the top-left corner of the linewidth channel.

## Source data

Source Data Fig. 2 Datapoints for the plots in Fig. 2b,c,d.

Source Data Fig. 3 Experimental datapoints shown as stars in Fig. 3a,b.

## Data Availability

The raw datasets generated and/or analysed for this work are available at 10.5281/zenodo.14505687 (ref. ^32^). Source Data are provided with this paper.

## Code availability

The code underlying the simulations presented in this paper are available at https://github.com/prevedel-lab/FT-Brillouin-microscopy (ref. ^33^).
