# Supplementary material for: Full-field Brillouin microscopy based on an imaging Fourier-transform spectrometer
Source: Nat Photonics. 2025 Feb 20;19(5):494–501. doi: 10.1038/s41566-025-01619-y (PMC12058527; doi:10.1038/s41566-025-01619-y)
Supplement: Supplementary file 1 — Supplementary Notes 1–6 and Table 1. [file 41566_2025_1619_MOESM1_ESM.pdf]

nature photonics

Supplementary information https://doi.org/10.1038/s41566-025-01619-y

Full-field Brillouin microscopy based on an imaging Fourier-transform spectrometer

In the format provided by the authors and unedited

SUPPLEMENTARY INFORMATION

Table of Contents

Supplementary Notes 2
Supplementary Note 1: Definition of arbitrary and typical Brillouin spectra 2
Supplementary Note 2: Determination of the sign for the amplitude 3
Supplementary Note 3: Effect of a wrong sign estimate on the reconstructed spectrum 3
Supplementary Note 4: Phase profile and reference inside the FT-interferometer 4
Supplementary Note 5: Calculation of the lineshape in the orthogonal scattering geometry 5
Supplementary Note 6: Calculation of the experimental Brillouin cross-section in FTBM.. 6
Supplementary Tables 7
Supplementary Table 1: Combined effect of different noise sources on spectrometer performance 7
Supplementary References 7

Supplementary Notes

Supplementary Note 1: Definition of arbitrary and typical Brillouin spectra

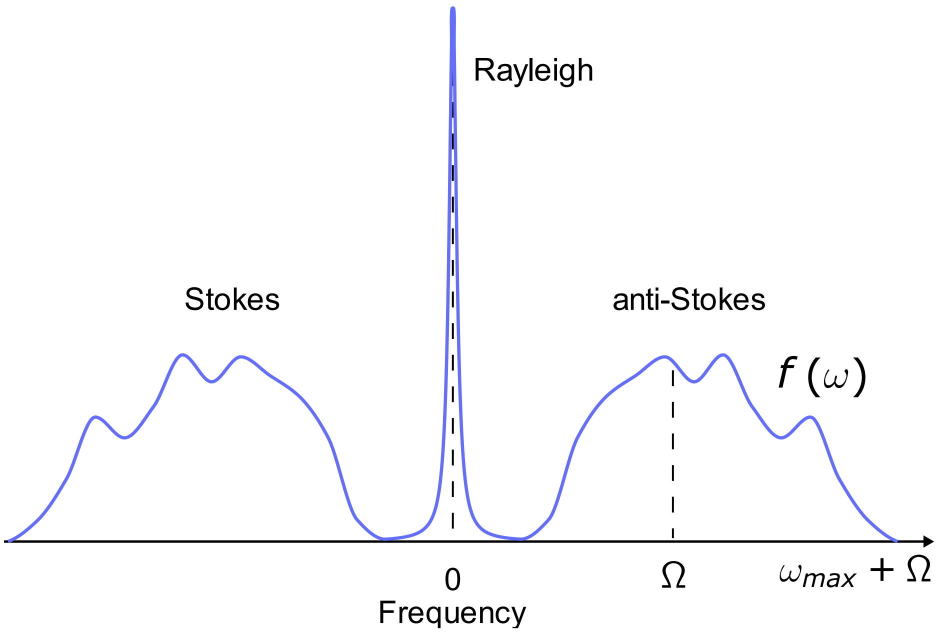

SI Figure 1.1: Schematic of an arbitrary symmetric spectrum. Note that $\omega_{max} + \Omega$ is the maximum permissible frequency (which can be reconstructed unambiguously).

For simplicity we assume that the optical spectrum has the general form of two symmetric sidebands (labelled as Stokes and anti-Stokes) on the side of a δ-like laser centered at the optical frequency ω_L (SI Fig. 1.1). Let's set $f(\omega) \in \mathbb{R}$ to be the anti-Stokes component of the spectrum, shifted by a frequency Ω from the laser line, and let's introduce the symmetric function $F(\omega) = f(\omega - \Omega) + f(-\omega - \Omega)$ which includes both the Stokes and anti-Stokes components. We can thus write the optical power spectrum as:

$$PS(\omega) = I_L \delta(\omega - \omega_L) + F(\omega - \omega_L) \quad (S1.1)$$

where I_L is the intensity of the laser and δ is the Dirac-delta.

According to the Wiener–Khinchin theorem:

$$\langle E(t)E(t-\tau)\rangle_t = Re\{\mathcal{F}\{PS(\omega)\}\} \quad (S1.2)$$

where $Re\{z\}$ is the real part of the complex number z and $\mathcal{F}\{g(\omega)\} = \tilde{g}(\tau)$ is the Fourier transform of a generic function $g(\omega)$. Note that the real part is introduced to account for a single sided power spectrum (which is usually used to represent an optical spectrum).

If we introduce equation $(S1.1)$ in equation $(S1.2)$ we get:

$$\langle E(t)E(t-\tau)\rangle_t = \cos(\omega_L \tau)\left\{I_L + 2Re\left\{e^{-i\Omega\tau}\tilde{f}(\tau)\right\}\right\} \quad (S1.3)$$

We note that the information about $F(\omega)$ is fully contained in the term

$$A(\tau) \coloneqq I_L + 2Re\left\{e^{-i\Omega\tau}\tilde{f}(\tau)\right\} = I_L + 2Re\{\mathcal{F}\{f(\omega - \Omega)\}\} = I_L + 2 \cdot \tilde{F}(\omega) \quad (S1.4)$$

which is the envelope of the term oscillating at the optical frequency $\cos(\omega_L \tau)$. Importantly, $F(\omega)$ can be recovered by taking the inverse-FT of $A(\tau)$.

In the specific case of a typical Brillouin spectrum consisting of a Lorentzian peak with amplitude A, shift $\nu_B = \Omega/2\pi$ and linewidth (FWHM) $\Delta\nu_B$, $f(\omega) = \frac{A}{1+\left(\frac{\omega/\pi}{\Delta\nu_B}\right)^2}$, $\tilde{f}(\tau) = Ae^{-\pi|\tau|\cdot\Delta\nu_B}$ and equation $(S1.4)$ becomes:

$$A(\tau) = I_L + 2Ae^{-\pi|\tau|\cdot\Delta\nu_B}\cos(\Omega\tau) \quad (S1.5)$$

Supplementary Note 2: Determination of the sign for the amplitude

From equation (S1.4) it follows that $A(\tau)$ can be negative when the optical power of the laser I_L is smaller than the power of the signal of interest (which is desirable). As shown in Supplementary Note 3, the knowledge of the sign is essential for the proper reconstruction of the spectrum. Experimentally, the effect of a negative sign of $A(\tau)$ is a phase shift of π in the $\cos(\omega_L \tau)$ term of the interferogram in equation (S1.3). Therefore, our strategy is to compute the difference between the phase expected in case of monochromatic light and the measured phase; if the absolute value of the difference is π the sign is considered to be negative. Due to the noise the phase difference will not be exactly 0 or π, therefore we use $\pi/2$ as a threshold. But how to determine the theoretical phase? In principle one could calculate it from the wavelength and the position of the stage. The wavelength is known with extremely high accuracy (<0.1pm), since the laser is locked to the Rb reference, but the accuracy on the stage position needs to be ~10nm, which is challenging to obtain over the full travel range (>100mm). To lessen the requirements on the stage precision/accuracy, we use an internal reference: we introduce a small portion of the main laser at the edge of the FOV in the intermediate image plane (see Extended Data Fig. 1). Such reference beam has a fixed phase relationship with the other spatial points, which is calculated in Supplementary Note 4 and can be experimentally characterized by acquiring the interferogram from a highly scattering sample (or by removing the Rb cell). Note that the characterization needs to be performed only once, if the alignment of the interferometer is not changed. The difference between the phase of each spatial point and the reference beam can then be compared with the "ideal" phase. SI Fig. 2.1 visually summarizes the described algorithm.

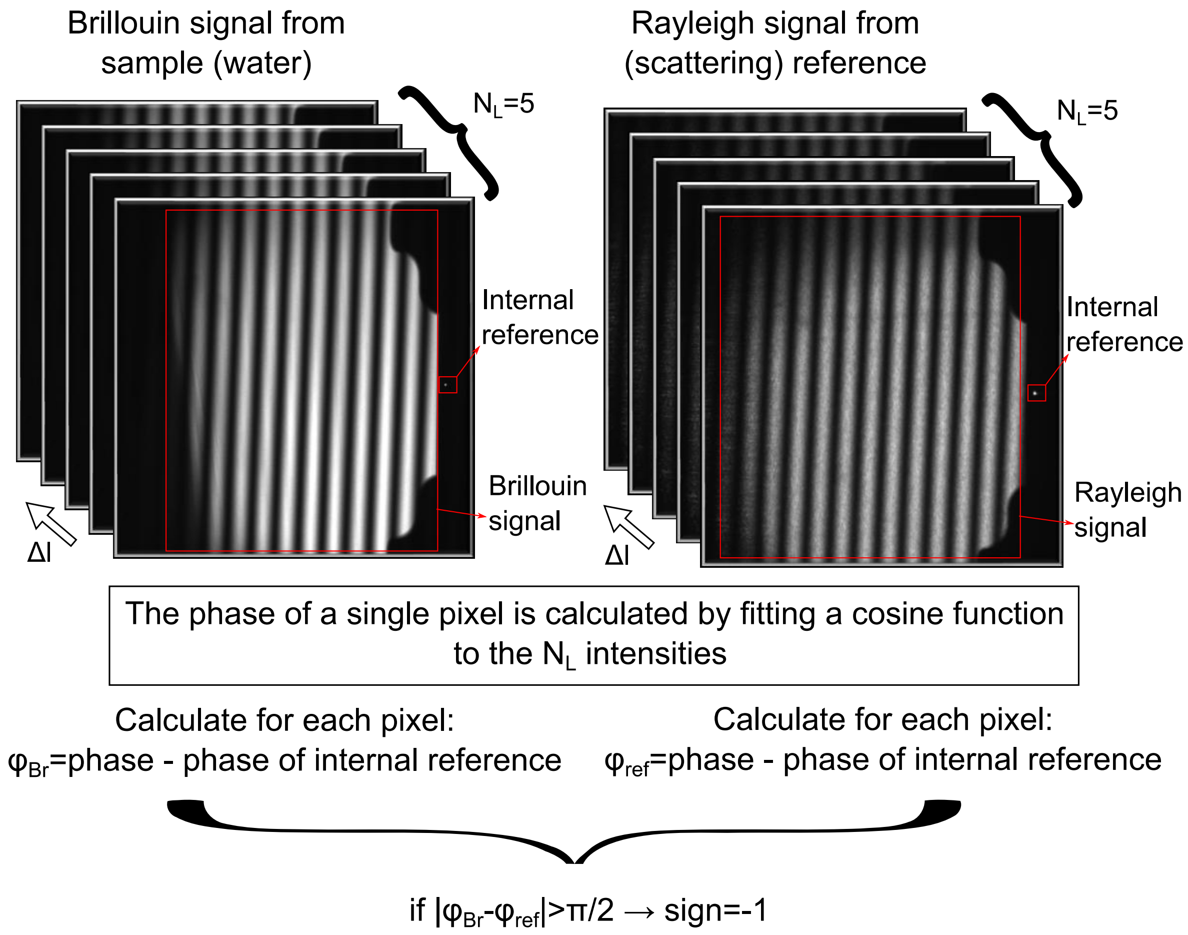

SI Figure 2.1: Summary of the procedure used to reconstruct the phase from the raw interferogram data. See Supplementary Note 2 for details.

Supplementary Note 3: Effect of a wrong sign estimate on the reconstructed spectrum

Let's assume we sample the amplitude of the envelope $A(\tau)$ at discrete τ_n, $n = \{0, \dots, N-1\}$. We can model a sign flip at the n_0-th sample by multiplying $A[\tau_n]$ by $flip_{n_0}[n] = 1 - 2\delta[n - n_0]$, where $\delta[n]$ is the Kronecker delta:

$$A_{flip}[\tau_n] = A[\tau_n] \cdot flip_{n_0}[n] = A[\tau_n] \cdot \{1 - 2\delta[n - n_0]\} \quad (S3.1)$$

Since the power spectrum $PS(\omega)$ is the inverse-FT of $A(\tau)$ (see Supplementary Note 1), we have:

$$PS_{flip}(\omega) = \mathcal{F}\{A_{flip}\} = PS(\omega) \otimes \mathcal{F}\{flip\}$$

where $\mathcal{F}\{\cdot\}$ represents the Fourier Transform and $\otimes$ the convolution.
Given the linearity of the convolution, we only need to calculate the term $\mathcal{F}\{A[\tau_n] \cdot \delta[n - n_0]\}$, which is easiest done by using the definition of the discrete inverse Fourier Transform and the Kronecker delta:

$$\mathcal{F}\{A[\tau_n] \cdot \delta[n - n_0]\} = \frac{1}{2N} \sum_{n=-N+1}^{N-1} A[\tau_n] \cdot \delta[n \pm n_0] \cdot e^{i\omega n} = A[\tau_{n_0}]/N \cdot \cos(\omega n_0)$$

where the symmetry of $A(\tau)$ was taken into account. It follows that the reconstructed power spectrum after a sign flip is:

$$PS_{flip}(\omega) = PS(\omega) - 2A[\tau_{n_0}]/N \cdot \cos(\omega n_0)$$

If multiple sign flips are present, equation (S3.1) needs to be multiplied by additional flip terms. Let's calculate the product of two flip terms at n_0 and n_1:

$$flip_{n_0} \cdot flip_{n_1} = \{1 - 2\delta[n - n_0]\} \cdot \{1 - 2\delta[n - n_1]\} = 1 - 2\delta[n - n_0] - 2\delta[n - n_1]$$

where the cross-term $\delta[n - n_0] \cdot \delta[n - n_1]$ is 0 due to the definition of the Kronecker delta.
Therefore, the general expression when L sign flips are present at n_i, $i = \{0, \ldots, L - 1\}$ is:

$$PS_{flip}(\omega) = PS(\omega) - \frac{2}{N} \sum_{i=0}^{L-1} A[\tau_{n_i}] \cdot \cos(\omega n_i)$$

From the previous equation we can infer that the effect of a sign flip at n_i is the addition of a sinusoidal background to the spectrum whose amplitude is small when $A[\tau_{n_i}] \approx 0$. This is important since, experimentally, points with small amplitude have higher noise (especially in the phase), which could lead to wrong sign flips.

Supplementary Note 4: Phase profile and reference inside the FT-interferometer

To aid in the optical design of the FT imaging spectrometer, it is useful to calculate the spatial map of the phase as a function of the optical path difference. Since the Michelson is in the infinity space, each lateral position in the sample corresponds to a collimated beam with a different angle with respect to the optical axis, thus undergoing a difference optical path inside the interferometer. Below we derive an expression for the optical path difference (OPD) between the two arms (SI Figure 4.1) as a function of the relative axial displacement between the retroreflectors ΔL, the relative lateral displacement of the retroreflectors due to not perfect alignment δ and the angle between the beam and the optical axis θ.

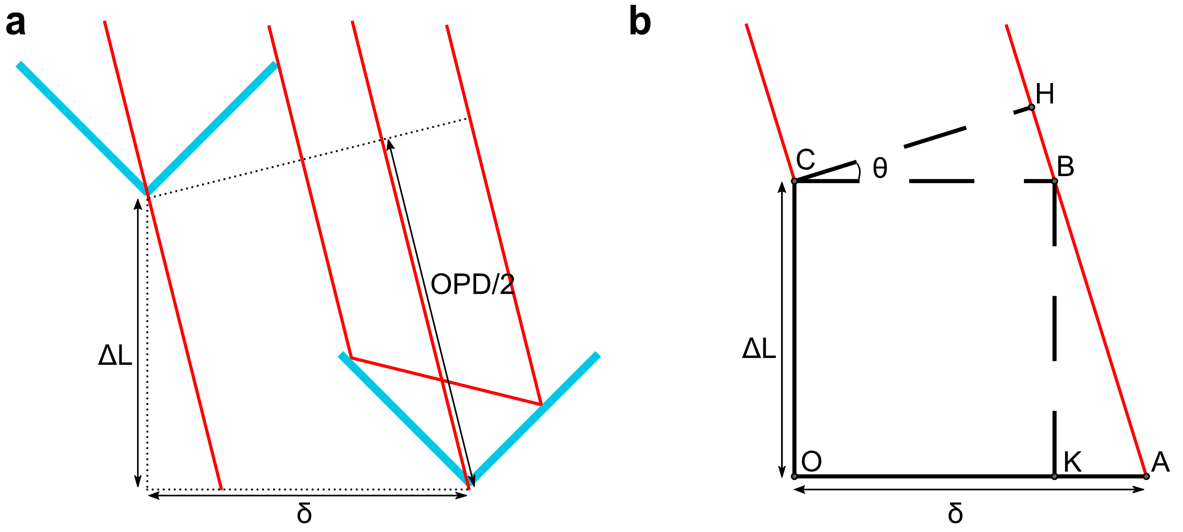

SI Figure 4.1: Schematic of the optical path of the rays inside the interferometer. a, Schematic of the two retroreflectors (light blue) and optical rays going through the vertex (dark red), with the relevant distances defined. **b**, same as panel a, where the retroreflectors are not shown and auxiliary lines and labels are introduced to aid in the calculations.

The optical path for all the rays within the beam is the same as the one for the ray going through the vertex of the retroreflector.
All the following calculations are performed in the plane Σ defined by the optical axis and the ray going through the vertex of the first retroreflector. Under this assumption, δ correspond to the projection of the physical

displacement $\delta_{TOT} = (\delta_x, \delta_y)$ on Σ, i.e. $\delta = \delta_{TOT} \cdot \cos\Phi$, where Φ is the angle between the Σ and the horizontal (x) plane.

$$\overline{CB} = \overline{OA} - \overline{KA} = \delta - \Delta L \tan\theta$$
$$\overline{BH} = \overline{CB} \sin\theta = (\delta - \Delta L \tan\theta)\sin\theta$$
$$\overline{AB} = \frac{\overline{BK}}{\cos\theta} = \frac{\Delta L}{\cos\theta}$$
$$\overline{AH} = \overline{AB} + \overline{BH} = \Delta L\left(\frac{1}{\cos\theta} - \frac{\sin^2\theta}{\cos\theta}\right) + \delta\sin\theta = \Delta L\cos\theta + \delta\sin\theta$$

In the approximation of $\theta \ll 1$ (θ< 0.02 rad in our experimental realization):

$$\overline{AH} \approx \Delta L\left(1 - \frac{\theta^2}{2}\right) + \delta \cdot \theta$$

The angle θ corresponds to the spatial position on the camera (x_c, y_c) (where the intersection of the optical axis with the sensor is set to (0,0)) through the relationship $\theta \approx \frac{r}{f}$, where $r := \sqrt{x_c{}^2 + y_c{}^2}$ and f is the focal length of the tube lens. Therefore, in terms of the coordinates of the camera:

$$\overline{AH} \approx \Delta L\left(1 - \frac{r^2}{2f^2}\right) + \delta\frac{r}{f} = -\frac{\Delta L}{2f^2}r^2 + \frac{\delta}{f}r + \Delta L$$

Since $\delta = \delta_{TOT} \cdot \cos\Phi$, $\delta \cdot r$ is the projection of δ_{TOT} on the direction of r and it can thus be written as the inner product $\delta_x \cdot x_c + \delta_y \cdot y_c$.

Finally, the OPD is:

$$OPD = 2\overline{AH} = -\frac{\Delta L}{f^2}(x_c{}^2 + y_c{}^2) + 2\frac{\delta_x \cdot x_c + \delta_y \cdot y_c}{f} + 2\Delta L$$

Given a fixed position of the retroreflector (ΔL=const), each spatial position on the camera corresponds to a beam that underwent a different optical path inside the interferometer, thus generating fringes. The spatial map of the phase of the fringes is given by:

$$\varphi(x_c, y_c) = \varphi_0 + k \cdot OPD = \varphi'_0 + \frac{2\pi}{\lambda f}\left[-\frac{\Delta L}{f}(x_c{}^2 + y_c{}^2) + 2(\delta_x \cdot x_c + \delta_y \cdot y_c)\right]$$

The period of the fringes Λ (i.e. the distance over which the phase changes by 2π) is a linear function of x_c and y_c, since $\varphi(x_c, y_c)$ is quadratic in x_c, y_c. For simplicity we calculate Λ only for the x direction, since the expression for the y direction is equivalent. We can calculate it by imposing that the $|\varphi(x_{c,2}) - \varphi(x_{c,1})| = 2\pi$ and assuming $x_c \approx \frac{x_{c,1}+x_{c,2}}{2}$

$$\Lambda = \frac{\lambda f^2}{2|-\Delta L \cdot x_C + f \cdot \delta_x|}$$

We must make sure that the pixel size on the camera Δx_C is sufficiently small to sample the fringes properly, i.e. $\Delta x_C < \Lambda/4$. For the following calculation we assume $\delta_x = 0$ (i.e. perfect alignment).

$$\Delta x_C < \frac{\Lambda}{4} = \frac{\lambda f^2}{8\Delta L \cdot x_C} < \frac{\lambda f^2}{8\Delta L_{max} \cdot x_{C,max}}$$

$x_{C,max} = M_{eff} \cdot FOV/2$ where M_{eff} is the overall magnification from the sample plane to the camera and FOV is the field of view on the sample plane.

$$\Delta x_C < \frac{\lambda f^2}{4\Delta L_{max} \cdot M_{eff} \cdot FOV}$$

In our design M_{eff} = 14.25, $FOV \approx$ 350mm and ΔL_{max} = 100mm, thus $\Delta x_C <$ 25µm, which is compatible with the pixel size of our camera (6.5µm).

Supplementary Note 5: Calculation of the lineshape in the orthogonal scattering geometry

In this note we aim to calculate, under the approximation of medium NA, an analytical expression for the lineshape of a single Brillouin peak in the geometry where the incident and scattered chief rays form an angle of 90 degrees.

Let's introduce ψ as the scattering angle and $\nu_\pi = \Omega_\pi/2\pi$ and $\Delta_\pi = \Gamma_\pi/2\pi$ as the Brillouin shift and linewidth (FWHM) in a backscattering geometry ($\psi = \pi$). The Brillouin shift and linewidth at a generic ψ are given by Ref.[1]:

$$\Omega = \Omega_\pi \sin(\psi/2)$$
$$\Gamma = \Gamma_\pi \sin^2(\psi/2)$$

Assuming a Lorentzian lineshape, the time domain signal at the output of the interferometer reads:

$$A_\psi(\tau) = \exp\left\{-\left[\frac{\Gamma_\pi}{2}\sin^2\left(\frac{\psi}{2}\right)|\tau| + i\Omega_\pi\sin\left(\frac{\psi}{2}\right)\tau\right]\right\}$$

Due to the finite NA of illumination and detection, the scattering angle ψ assumes different values from a distribution $f(\psi)$ and the measured Brillouin signal is thus:

$$A(\tau) = \int f(\psi) \cdot A_\psi(\tau) d\psi$$

In our experimental conditions $f(\psi)$ is mainly determined by the detection NA (0.8), thus ψ is the range $\frac{\pi}{2} - \sin^{-1}\frac{0.8}{1.33}$ and $\frac{\pi}{2} + \sin^{-1}\frac{0.8}{1.33}$. Expanding the terms $\sin(\psi/2)$ and $\sin^2(\psi/2)$ to the first order at $\psi = \pi/2$ introduces an error smaller than 5%. Under this approximation and assuming $f(\psi)$ is a normal distribution, we can calculate an analytical expression for the previous integral:

$$A(\tau) = \exp\left[-\frac{\Gamma_{\pi/2}}{2}|\tau| - \frac{\sigma^2}{2}\tau^2\left(\Omega_{\pi/2}^2 - \Gamma_{\pi/2}^2\right)\right]\cos\left(\Omega_{\pi/2}\tau - \frac{\sigma^2}{2}|\tau|\tau \cdot \Gamma_{\pi/2}\Omega_{\pi/2}\right) \quad (S5.1)$$

where σ is half of the standard deviation of the distribution $f(\psi)$ and we have introduced $\Gamma_{\pi/2} = \Gamma_0/2$ and $\Omega_{\pi/2} = \Omega_0/\sqrt{2}$. Note that, when $\sigma = 0$, the previous equation is consistent with equation S1.5.
To determine σ we took the reference values for Brillouin shift and width of water at our wavelength from literature and we fitted the experimental spectra of water with equation S5.1 keeping $\Omega_{\pi/2}$ and $\Gamma_{\pi/2}$ fixed to the reference values while leaving σ as a free parameter. We found $\sigma \approx 0.18$ corresponding to an effective NA of ~0.47 ($1/e^2$). The discrepancy with our nominal value (0.8) is likely due to a loss in NA caused by non-optimal optics on the detection side, as well as due to the assumption of a distribution (Gaussian) which doesn't fully represent the experimental data. Nevertheless, we note that this approximated calculation predicts well the experimental linewidth and shape.
Furthermore, when the quadratic term in the phase of equation S5.1 can be neglected (i.e. $\sigma^2 \ll 2/(\tau \cdot \Gamma_{\pi/2})$), the corresponding lineshape in frequency domain is a Voigt profile, allowing to calculate the FWHM from the fit parameters[2].

Supplementary Note 6: Calculation of the experimental Brillouin cross-section in FTBM

In the simulation (Fig. 3) we report the number of detected photoelectrons rather than input power/dwell time, since the latter is dependent on the actual implementation of the FTBM (pixel size, lightsheet width, etc...). In this Supplementary Note we aim to give an estimate of the number of detected photoelectrons given the experimental parameters.

If we assume that the intensity of light along the width of the lightsheet is constant (top hat) and the lightsheet is imaged on N_x pixels on the camera, each pixel on the camera "sees" an input power of:

$$P_i \approx P_{TOT}/N_x$$

where P_{TOT} is the total input power and no depletion of the input power along the lightsheet is assumed (weak absorption/scattering).
If the signal is integrated over a dwell time Δt, the total number of input photons per pixel is given by:

$$N_i \approx \frac{P_i \cdot \Delta t \cdot \lambda}{hc} \approx 5.03 \cdot 10^{24}[\mathrm{J}^{-1}\mathrm{m}^{-1}] \cdot P_i \cdot \Delta t \cdot \lambda$$

Assuming all the Brillouin photons scattered across the thickness of the lightsheet are collected and introducing μ as the "scattering coefficient" for Brillouin scattering (i.e. $\mu \cdot l \cdot N_i$ Brillouin photons are scattered over a distance l), we can find the total number of Brillouin scattered photons:

$$N_{Br} = \mu \cdot p \cdot N_i$$

where p is the pixel size on the sample plane.
Finally, considering the quantum efficiency QE of the camera, we arrive at the number of detected photoelectrons per pixel:

$$N_{detect} = QE \cdot N_{Br} = 5.03 \cdot 10^{24}[\mathrm{J}^{-1}\mathrm{m}^{-1}] \cdot QE \cdot P_{TOT}/N_x \cdot \Delta t \cdot \lambda \cdot p \cdot \mu$$

From SI Fig. 6.1a, we can derive that for our implementation ($N_x = 800$) $\mu \cdot p \cdot QE = 3.4 \cdot 10^{-12}$; given $QE \approx 55\%$ and $p \approx 0.44$ µm, we find $\mu \approx 1.4 \cdot 10^{-11}$ µm^{-1}, which is consistent with the expected cross section of Brillouin scattering in water.

Finally, to provide some guidance in determining the required input power/dwell time for a desired precision in the Brillouin shift/width, we summarized our experimental data on water in SI Fig. 6.1b. Note that to calculate the total input energy on the sample, one has to multiply the energy per pixel by the number of pixels in our FTBM image (i.e., ~400.000 as reported in the caption of Extended Data Fig. 3). For example, in order to acquire a 2D image with a shift precision of ~20MHz, the total energy on the sample needs to be ~0.01mJ/px x 400.000px ≈ 4 J.

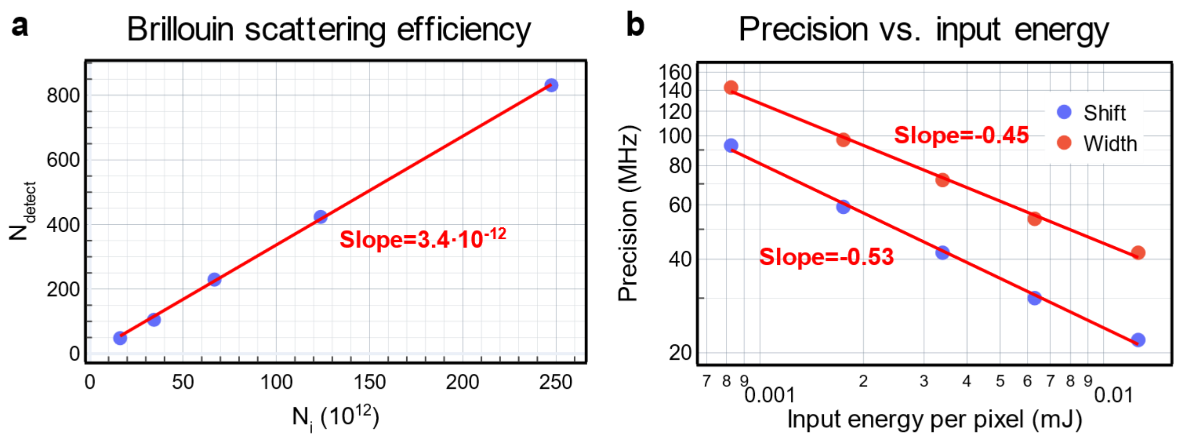

SI Figure 6.1: Experimental data for the conversion of illumination energy to number of Brillouin photons and precision. a, Plot of number of detected photoelectrons on the camera against the number of incident photons on each "pixel" on the sample plane; the slope of a linear fit represents the efficiency of the scattering process and collection. **b,** Plot of Brillouin shift and width precision against the input energy per pixel (calculated as optical power per pixel x dwell time x number of interferometer samples).

Supplementary Tables

Supplementary Table 1: Combined effect of different noise sources on spectrometer performance

To illustrate the combined effect of additional noise sources, this table lists the expected (simulated) precision of the FTBM spectrometer based on exemplary parameters while keeping the number of photoelectrons fixed. In parenthesis we report the precision in MHz (shift/width) due to the single source. We note that different combination of noise sources and their amplitude can be simulated using our provided code.

Number of photoelectrons	Camera noise (e-)	Stage precision (nm)	Rayleigh intensity	Intensity noise (%)	Combined effect on precision (MHz)
100 (53±2/147±7)	1.4 (10.2±0.2/28±1)	10 (0/0)	0.01 (0/0)	0.2 (0.74±0.03/2.2±0.1)	57±2/153±4
100 (55±3/146±6)	5 (38±1/105±4)	22 (0/0)	1 (0/0)	0.6 (2.2±0.1/6.7±0.2)	86±4/250±10
100 (54±3/145±8)	5 (37±2/102±3)	49 (0/0)	2 (0/0)	2 (7.7±0.3/23.9±0.8)	161±20/449±108

Supplementary References

1. Antonacci, G., Foreman, M. R., Paterson, C. & Török, P. Spectral broadening in Brillouin imaging. *Appl. Phys. Lett.* **103**, 5–8 (2013).
2. Olivero, J. J. & Longbothum, R. L. Empirical fits to the Voigt line width: A brief review. *J. Quant. Spectrosc. Radiat. Transf.* **17**, 233–236 (1977).
